# Ultrahigh‐Linear Bio‐Inspired Janus Elastomeric Strain Sensor with High Sensitivity and Stretchability via Surface Wrinkle Engineering

**DOI:** 10.1002/advs.202524269

**Published:** 2026-01-27

**Authors:** Jing Lin, Ye Li, Simi Yu, Xing Cheng, Longxin Qian, Yufei Chen, Womin Li, Zhipeng Yang, Yinlei Lin, Dechao Hu, Jianyi Luo, Lan Liu

**Affiliations:** ^1^ Research Center of Flexible Sensing Materials and Devices School of Applied Physics and Materials Wuyi University Jiangmen China; ^2^ School of Materials and Energy Foshan University Foshan China; ^3^ Key Lab of Guangdong High Property and Functional Macromolecular Materials School of Materials Science and Engineering South China University of Technology Guangzhou China

**Keywords:** biomimetic design, crack mechanism, high linearity, Janus structure, strain sensor

## Abstract

In pursuit of high‐performance flexible strain sensors, achieving an optimal trade‐off among linearity, sensitivity, and strain sensing range remains a critical challenge. Inspired by the wrinkled‐leaf viburnum, we develop a Janus sensor that replicates its asymmetric structure. It comprises a dense, micro‐wrinkled natural rubber (NR)/graphene (GRs) top layer and a loose NR/carbon nanotubes (CNTs) bottom layer, fabricated via facile layer‐by‐layer filtration and pre‐stretching strategy. This bio‐inspired design enables the sensor with a synergistic sensing mechanism: wrinkle‐guided microcrack ensures highly sensitive linear response at low strains; strain‐phase division maintains signal continuity at medium strains; and parallel conductive circuits provide robustness at high strains. As a result, the sensor achieves an exceptional combination of ultra‐high linearity (R^2^ > 0.999) and sensitivity (gauge factors, GF > 14) across 0–100% strain, with a wide sensing range (> 400%) and fast response (0.16 s). We demonstrate its practical value in human motion detection, physiological signal monitoring, and an intelligent glove system for gesture recognition and human‐machine interaction, highlighting its promising potential for advanced wearable devices and human‐machine interactive systems.

## Graphical abstract

A bio‐inspired Micro‐wrinkled Janus elastomeric flexible strain sensor mimicking wrinkled‐leaf viburnum is developed. Endowed with a synergistic sensing mechanism including wrinkle‐guided microcracks, modulus‐gradient‐driven strain division, and a parallel conductive circuit, it achieves ultra‐high linearity, sensitivity, wide strain range, and excellent cyclic stability, showing great potential in wearable electronics and human‐machine interaction.

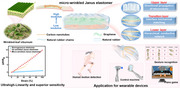

## 1 Introduction

Flexible strain sensors, capable of converting mechanical deformations such as tension, compression, and bending into measurable electrical signals, are pivotal for advancements in intelligent robotics, wearable electronics, and human‐machine interfaces [1, 2, 3]. For wearable applications, strain sensors must possess high sensitivity, high linearity, and high sensing range (>50%) to accommodate multiscale and dynamic deformations induced by human activities. Among them, a linear response which can simplify signal calibration, reduce wearable device algorithm complexity, and ensure consistent long‐term measurement accuracy is of great significance. To date, elastomer‐based strain sensors have attracted significant interest owing to their facile fabrication, excellent stretchability, and superior skin conformability [4, 5]. However, optimizing sensitivity, linearity, and sensing range simultaneously poses a key challenge, hindering the accuracy and practical application of strain sensors.

This inherent performance trade‐off originates from the competing dynamic behaviors of the conductive network under external stimuli. High sensitivity in flexible strain sensors typically originates from the irreversible destruction of conductive networks, which usually works only within a narrow sensing range and exhibits non‐linear resistance changes [6, 7]. For example, Liu et al. developed a molecular‐level crack‐based sensor that exhibited an ultra‐high gauge factor (∼500 at 0.001% strain), yet this high sensitivity was accompanied by signal nonlinearity (ultranarrow range of 0–20%) and a constrained sensing range (up to 35%) [8]. Conversely, achieving a wide sensing range requires a robust, often high‐filler‐loading network that can undergo reversible deformation. Such designs usually demonstrate limited sensitivity (GF < 10) and a wide linear range [9, 10]. The intrinsic limitations include: (i) the conflict between irreversible brittle failure for high sensitivity and reversible deformation for large sensing range; (ii) mechanical mismatch at heterogeneous interfaces leading to stress concentration and poor durability; and (iii) the mutual exclusivity between low‐percolation‐threshold networks for high sensitivity and stable networks for wide sensing range [11, 12, 13].

In this regard, constructing Janus heterogeneous conductive networks has been a primary strategy [14, 15]. It has been reported that strategies employing gradient fillers, sparse‐dense distributions, or asymmetric conductive networks between upper and lower layers can resolve strain responses across different ranges, thereby enhancing sensitivity over a wide strain [16, 17, 18]. On the other hand, introducing microstructures like wrinkles or pores can guide crack propagation and alleviate stress concentration, thereby improving linearity [19, 20, 21]. For instance, Janus heterostructured microarrays and dual‐conductive‐layer sensors with wrinkles have achieved enhanced sensitivity and linearity. Shao et al. successfully fabricated a Janus heterostructured microarray strain sensor with silver nanoparticles in situ grafted in a micropatterned elastomeric film, achieving a GF >10 and high linearity within 55% strain [15]. Similarly, Wu et al. proposed a GRs/CNTs/Polydimethylsiloxane (PDMS)‐based strain sensor with a dual‐conductive layer and a wrinkled structure, which provided a linearity of 0.999 up to 30% strain [22]. Notably, in both cases, the high linearity (R^2^ > 0.999) is constrained below approximately 55% strain, as the microstructures lose their modulating function once fully flattened. Furthermore, the fabrication of such sophisticated heterostructures often involves complex and costly processes, hindering their scalable application.

**FIGURE 1:**
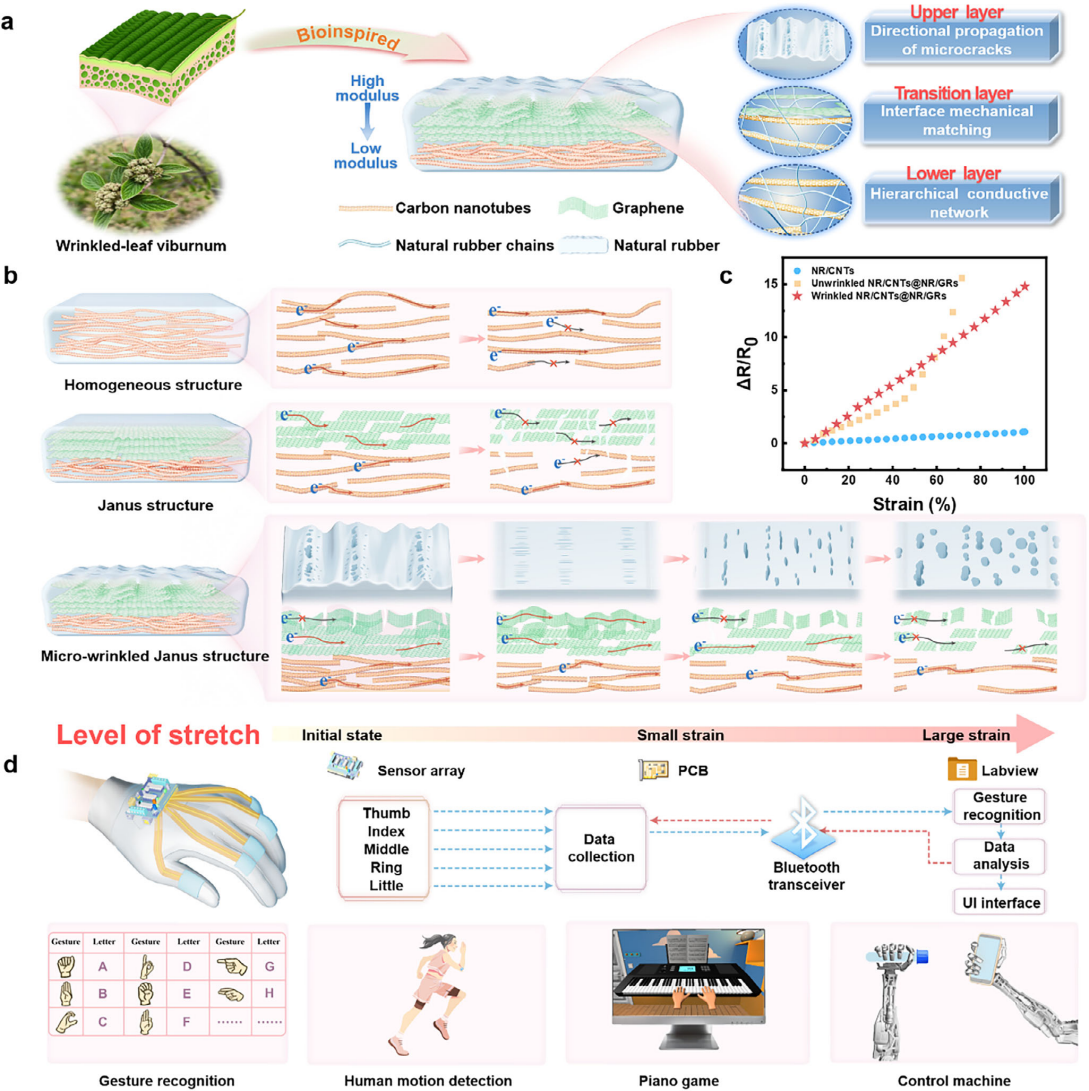
Design principle and application of micro‐wrinkled Janus film. (a) Schematic illustration of wrinkled‐leaf viburnum and bio‐inspired Janus film with micro‐wrinkle structure. (b), (c) The schematic diagram of electron transport pathways and electrical signal response of NR/CNTs, unwrinkled NR/CNTs@NR/GRs, and micro‐wrinkled NR/CNTs@NR/GRs films. (d) The application of micro‐wrinkled Janus film‐based sensors in human motion motoring and human‐computer interaction.

Nature provides ingenious solutions to such engineering challenges. Wrinkled‐leaf viburnum, as shown in Figure 1a, possesses a remarkable Janus microstructure. This natural bionic prototype features a rigid, wrinkled front side (enriched with cellulose, ensuring high modulus) and a soft, porous back side (composed of flexible parenchyma cells, enabling excellent deformability), interconnected by a gradient transition layer that relieves interface stress. This asymmetric and graded architecture enables strain‐phase division and guided crack propagation, where the rigid layer bears load, and the soft layer adapts to deformation, thereby allowing the leaf to maintain mechanical integrity across a wide strain range.

Inspired by this natural design, we propose a Janus wrinkled elastomeric sensor fabricated via a facile layer‐by‐layer vacuum filtration and pre‐stretching strategy. This design features an asymmetric double conductive network, composed of an NR/GRs layer and an NR/CNTs layer, and an ordered wrinkle architecture, replicating the key morphological features of the leaf (Figure 1a). Specifically, the rigid front side of the viburnum leaf directly corresponds to the NR/GRs upper layer, where graphene not only endows high modulus analogous to leaf cellulose but also provides excellent electrical conductivity. In contrast, the soft back side of the leaf matches the NR/CNTs lower layer, where dispersed CNTs ensure moderate flexibility similar to parenchyma cells while maintaining conductive network continuity. By replicating the gradient transition layer of natural leaves via vulcanization, the sensor achieves robust interfacial integration and relieves stress concentration between the two layers. This bionic structure enables the sensor with a synergistic sensing mechanism (Figure 1b): at low strains (0–100%), wrinkle‐guided microcracks provide linear and high‐sensitivity response; at medium strains (100–200%), modulus‐gradient‐driven strain division ensures smooth signal transition; and at high strains (> 200%), a parallel conductive circuit maintains network integrity for wide‐range operation. As a result, the sensor achieves an ultra‐high linearity (R^2^ > 0.999) and high sensitivity (GF > 14) across 0–100% strain, while maintaining a sensing range exceeding 400% and a low detection limit of 0.1%. It also exhibits fast response/recovery (0.16/0.16 s) and excellent cyclic stability. Compared to the homogeneous NR/CNTs and unwrinkled NR/CNTs@NR/GRs sensors, this sensor exhibits more wider linear response and demonstrates a significant advantage over existing strategies (Figure 1c). These attributes enable reliable performance in human motion detection, intelligent perception, and human‐machine interaction, highlighting its potential for smart wearable electronics (Figure 1d).

## 2 Results and Discussion

### 2.1 Structure and Mechanical Properties

**FIGURE 2:**
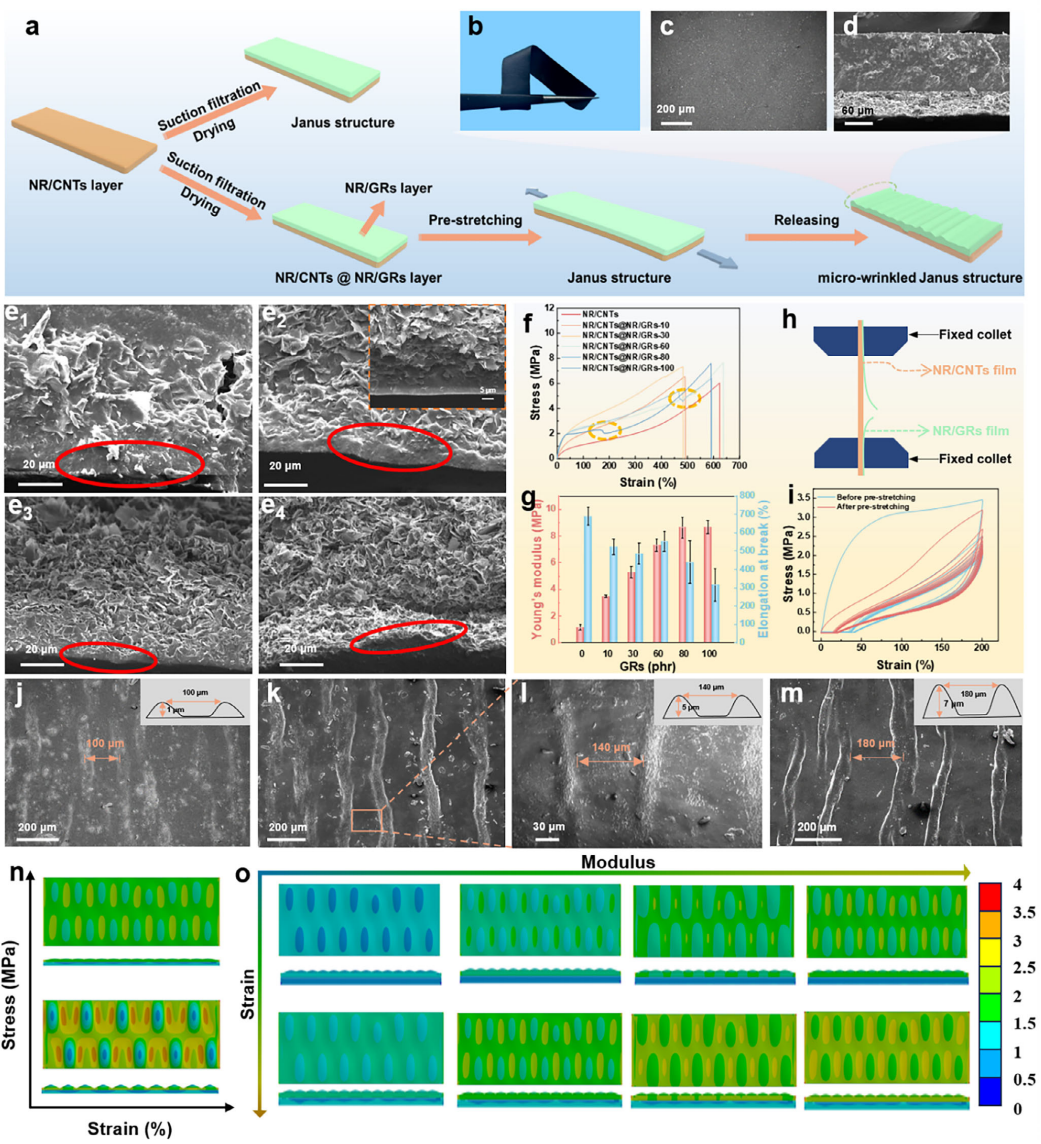
Preparation, structural characterization, and mechanical properties of the micro‐wrinkled Janus structured NR/CNTs@NR/GRs film. (a) Fabrication schematic illustration of the micro‐wrinkled Janus film. (b–d) Photographs, surface‐section, and cross‐section SEM of the micro‐wrinkled Janus film with NR/CNTs and NR/GRs layers. (e) Cross‐section SEM of the NR/GRs layer for the micro‐wrinkled Janus film. (f), (g) Typical stress–strain curves and the corresponding mechanical properties of the micro‐wrinkled Janus film with varying GRs contents. (h) Schematic diagram of the NR/GRs layer fracture for the micro‐wrinkled Janus film. (i)Stress–strain hysteresis loop curves under five cycles of the micro‐wrinkled Janus film before and after pre‐stretching of 200% strain. (j–m) SEM images of the micro‐wrinkled Janus film with GRs of 60 phr at 100%, 200%, and 300% pre‐stretching strain. ANSYS mechanical simulation for stress distribution of the micro‐wrinkled Janus film with different content of wrinkles (n) and various GRs contents (o).

The micro‐wrinkled Janus structured film composed of a low‐filler conductive layer (NR/CNTs) and a high‐filler conductive layer (NR/GRs) was fabricated via layer‐by‐layer vacuum filtration (Figure 2a, Figure S1). Subsequently, the assembled film was fully vulcanized through heating to form a robust transition layer, ensuring strong interfacial adhesion between the two conductive layers. A micro‐wrinkled structure was then induced in the NR/GRs layer by pre‐stretching due to the difference in modulus of the double layer. Finally, the film was connected by copper electrodes to form flexible strain sensors. As shown in Figure 2b, a distinct color contrast is observed between the light gray NR/GRs top layer and the dark black NR/CNTs bottom layer, attributed to the different conductive networks within the elastomer matrix. Besides, SEM images further demonstrate the hierarchical structure formed by the two conductive layers within the NR matrix (Figure 2c, d).

The thickness and morphology of the individual layers were modulated by the GR's filler content. Cross‐sectional SEM images demonstrate that the NR/CNTs layer (15 phr CNTs) has a consistent thickness of ∼131 µm, while that of the NR/GRs layer varies from about 61 to 148 µm depending on the GRs loading (Figure 2e_1_–e_4_). As the GRs content increased, the pure NR on top of the NR/GRs layer progressively diminished (the enlarged SEM image in Figure 2e_2_), nearly disappearing at 100 phr GRs (marked by a red circle in Figure 2e_1_–e_4_), indicating that a gradient structure is formed through vacuum filtration and gravity drive process. Besides, the TEM image of the NR/GRs layer clearly shows that the GRs are uniformly distributed within the NR matrix without agglomeration, with a preferential orientation parallel to the film surface (Figure S2a). For the NR/CNTs layer, the CNTs are uniformly and homogeneously dispersed, with no observable agglomeration (Figure S2b). These TEM results demonstrated that Triton X‐100 modification combined with filtration enables uniform dispersion and ordered orientation of conductive fillers within the NR matrix. Consequently, the gradient structure together with these filler characteristics endows the sensor with excellent mechanical properties.

The mechanical properties of the composite film were systematically evaluated. The Young's modulus of the Janus film (derived from the stress–strain curves in Figure 2f) increases progressively with increasing GR content, while its elongation at break generally decreases from 700% to 300%, which is consistent with many reported works (Figure 2g) [23, 24]. The NR/CNTs@NR/GRs film with 60 phr GRs has the optimized mechanical property, with the elongation at break of 550%, and tensile strength of 7 MPa. Significantly, for films with high GRs contents, the stress in their stress–strain curves drops sharply during deformation (the circled region in Figure 2f), which is attributed to the sudden fracture of the NR/GRs layer. As illustrated in Figure 2h, which depicts the tensile test setup for the bilayer composite structure, the NR/GRs layer undergoes preferential fracture under tensile loading. This premature fracture undermines the load‐bearing capacity of the film, which in turn leads to an abrupt decline in the stress resistance of the samples and ultimately exerts an adverse effect on the sensing range of the sensor. Nevertheless, the decreased hysteresis loop area can be observed in the stress–strain hysteresis loop diagram of the NR/CNTs@NR/GRs film after pre‐stretching of 200% strain, demonstrating the residual stress release and excellent elastic mechanical property after pre‐stretching (Figure 2i). The sensor also demonstrates the ability to withstand practical deformations such as stretching, bending, and twisting (Figure S3). The strong interfacial interaction between the fillers and NR is responsible for the excellent mechanical properties of the NR/CNTs@NR/GRs film. As shown in Figure S4a, the characteristic peaks of NR (ascribed to C‐H bending vibrations and polyisoprene skeleton vibrations in the 1000–1500 cm^−^^1^ region) show reduced intensity in the films, which arises from the constrained vibrational freedom of NR molecular chains due to tight interfacial interactions with GR, verifying the intimate combination between GR and the NR matrix [25, 26]. For the XRD patterns (Figure S4b), the films exhibit a broadened diffraction peak (at 2θ = 19.1) corresponding to the amorphous region of NR, alongside an enhanced GRs (002) peak at 2θ = 26.5° as GRs content increases [27, 28]. This suggests that GRs are uniformly dispersed in the NR matrix, not only restricts the crystallization of NR, but also promotes the formation of a loose crystalline network that facilitates stress buffering. Furthermore, the excellent interaction between NR/CNTs and NR/GRs layers via vulcanization endows the film with superior mechanical and sensing stability.

To have an insight into the micro‐wrinkled Janus structure in the NR/CNTs@NR/GRs film, its formation conditions were discussed in detail. It's well known that the evolution of the wrinkled morphology hinges on the pre‐stretching strain applied to the as‐prepared NR/CNTs@NR/GRs film during fabrication. As shown in Figure 2j–m, the surface of the NR/GRs layer (60 phr GRs) exhibits ordered wrinkles with a depth of 1–7±2 µm and space interval of 100–180±15 µm with increasing pre‐stretching strain (100%, 200%, and 300%), while the NR/CNTs layer maintains a smooth surface due to the elastic modulus difference between the two layers (Figure 2c). Besides, the wrinkles become more obvious as the GRs content increases (Figure S5). Larger pre‐strains yield more high wrinkles with irregular structures after film release, while smaller pre‐strains result in low and simpler wrinkles. Although increased wrinkle formation correlates with reduced elongation at break (Figure S6), it significantly enhances the linearity over a wide strain range (Figure S7). When evaluated at 100% strain, films pre‐stretched at 100%, 200%, and 300% strain demonstrate linearities of 0.971, 0.999, and 0.998, respectively (Figure S8). Therefore, more wrinkles do not equate to better performance. Only when the matching degree between wrinkling degree and strain regulation is optimal (wrinkles corresponding to 200% pre‐strain) can linearity be maximally enhanced. With small wrinkles (at 100% pre‐strain), the upper NR/GRs conductive network lacks sufficient strain‐sensitive structures, such that the proportionality between resistance change and strain under small strain is low, resulting in poor linear fitting. Excessive wrinkles cause the conductive network to undergo severe fragmentation under a 300% pre‐strain condition, making the processes of wrinkle flattening and crack propagation under strain lose controllability. Thus, the proportional regularity of resistance change declines, and linearity is reduced. However, wrinkles regulate the strain‐resistance relationship most precisely with moderate wrinkles (200% pre‐strain), which guides resistance to change proportionally via orderly structures without disrupting this proportionality due to excessive fragmentation, hence achieving the best linearity. Notably, a micro‐crack structure is observed in the wrinkle (the inset in Figure 2l). Pre‐stretching causes the high‐modulus layer to generate cracks and pores because it bears stress that exceeds its fracture threshold, where these defects form to relieve the accumulated stress. When the applied stretching load is removed, the material experiences irreversible plastic deformation and cannot recover its original morphology, thus leading to the permanent retention of cracks and pores within the matrix.

To clarify the mechanism of wrinkle‐guided microcracks in our designed structure, ANSYS mechanical simulation analysis and the related experiments were performed. First, the sparse wrinkle structure lacks sufficient stress‐dissipating units, so applied loads cannot be evenly dispersed (Figure 2n). This leads to higher stress concentration under the same loading condition, indicating that low wrinkle density limits the sample's stress‐bearing capacity. In contrast, high‐density wrinkle film displays light cyan/blue‐green tones, where the dense, ordered wrinkles provide more pathways for stress transfer, enabling uniform dispersion of the applied load. In addition, the simulation reveals a relatively uniform stress distribution in the low‐module film with low GRs content under identical pre‐strain (Figure 2o). For the film with high GRs content, stress was concentrated behind the wrinkles in the NR/GRs layer, which serves as the favorable evidence for wrinkles guiding directional crack propagation [29]. On one hand, the concave‐convex morphology of wrinkles disrupts the uniform stress distribution inside the composite, forming high stress concentration areas at the convex and concave regions. These areas provide a continuous driving force for crack propagation, activating internal microcracks and promoting their preferential extension along the high‐stress zones. On the other hand, the continuously arranged wrinkle units form a coherent high‐stress band, constructing a crack propagation channel and preventing random crack branching. Besides, higher applied strain enhances the stress‐guiding capability of ordered wrinkles in high‐modulus film, further reinforcing directional crack propagation. In contrast, low‐modulus film shows no obvious directional stress distribution under either strain, so its wrinkles barely contribute to guiding directional crack propagation [30, 31]. Morphological changes of cracks at different strains were also recorded using a Leica camera (Figure S9). The experimental results clearly demonstrate that cracks propagate in a directional manner, which is consistent with the simulation predictions. This experimental evidence establishes a direct link between theoretical simulations and practical crack behavior, effectively validating the proposed propagation mechanisms. Furthermore, the SEM images reveal that the crack morphology, initiation sites, and propagation patterns remain consistent across the tested temperature and humidity ranges (Figure S10). This indicates that the crack behavior of the material is insensitive to the environmental factors within the scope of our research, providing an experimental basis for evaluating its environmental adaptability. Therefore, the designed wrinkles are expected to achieve precise regulation of crack propagation paths, which is of great significance for flexible sensor design.

### 2.2 Sensing Mechanisms of Micro‐Wrinkled Janus Structure

To gain insight into the sensing mechanisms of the fabricated sensor, the electrical conductivity of the micro‐wrinkled Janus film was first investigated. As shown in Figure S11, the electrical conductivity is at a low level (∼ 0.1 S/cm) when the content of GRs is below ∼30 phr, which is ascribed to that no continuous conductive network is formed in the NR matrix. Electrons have to be transported via the tunneling effect or long‐distance hopping, such that slight changes in filler contact points under strain barely alter the overall conduction efficiency of the conductive paths significantly, thereby resulting in extremely low sensitivity. When the content of GRs is about 30 phr, the electrical conductivity sharply increases. This abrupt transition point corresponds to the percolation threshold [32, 33]. At this threshold, GRs form a continuous conductive pathway for the first time via point‐to‐point contact, thereby causing a sharp drop in electron transport resistance. This resistance reduction, in turn, leads to an explosive increase in electrical conductivity. Importantly, the conductive network at this stage enables resistance to change proportionally to strain (exhibiting excellent linearity), thus significantly enhancing the sensor's sensitivity [34, 35]. Above the percolation threshold, the conductive network is excessively dense and exhibits high rigidity, rendering it hard to be disrupted or reconfigured under strain. Typically, the conductivity of the film with 60 phr GRs reaches 3.36 S/cm. However, further increasing the GRs content promotes nanosheet aggregation, which restricts elastomer chain mobility and degrades mechanical properties (Figure 2f). Meanwhile, once the GR's content surpasses the percolation threshold, the electrical resistance of the composite undergoes only insignificant variations, which in turn results in reduced sensitivity. Additionally, the network's “over‐stability” makes it prone to linearity degradation. Combining the mechanical property, the sensing property of the micro‐wrinkled Janus sensor with GRs content of 60 phr could be optimized, which is also verified by the following property test.

**FIGURE 3:**
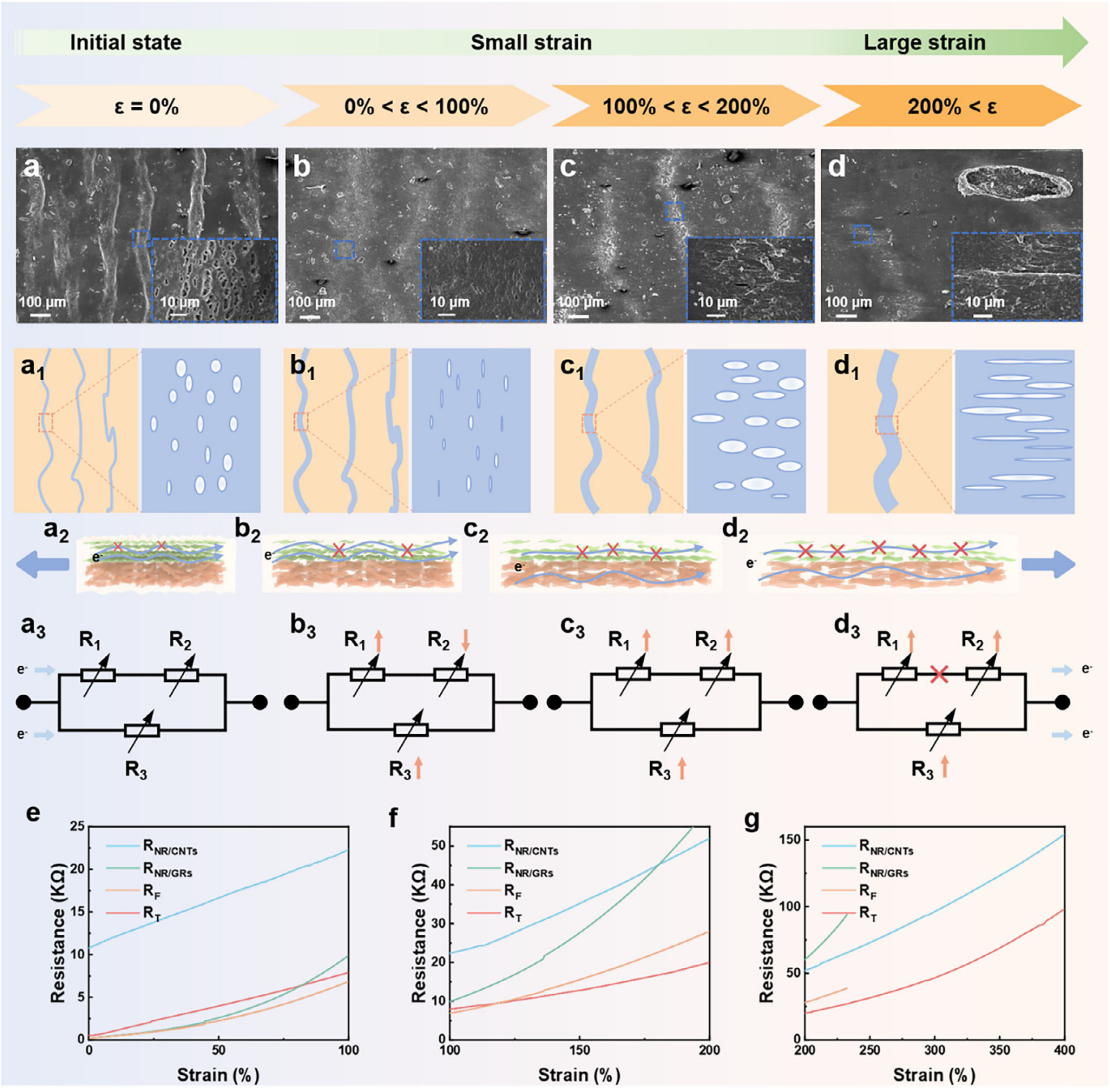
Sensing mechanism of the micro‐wrinkled Janus structured NR/CNTs@NR/GRs film. (a–d) SEM images of the sensor under different strains. (a_1_–d_1_), (a_2_–d_2_) Schematic diagram of the structure and conductive paths for the sensor under different strains. (a_3_–d_3_) The equivalent circuit diagrams of the sensor under different strains. (e–g) The resistance response of pure NR/CNTs, NR/GRs, the fitting parallel model (R_F_), and our designed micro‐wrinkled Janus structured NR/CNTs@NR/GRs film (R_T_).

The morphological evolution of the micro‐wrinkled Janus structure in film containing 60 phr GRs under applied strain was studied. It's well known that the sensing mechanism of a strain sensor involves the formation and propagation of cracks within the conductive network, where both cracks and wrinkles play a significant role in sensing performance [13, 14]. To clarify the underlying mechanism of micro‐wrinkled Janus structure, we propose an equivalent circuit model where the total resistance (R_T_) comprises parallel contributions from the resistance in the NR/GRs layer (R_1_: unwrinkled regions; R_2_: wrinkled regions) and the NR/CNTs layer (R_3_), as illustrated in the following formula (Figure 3a_3_):

$$R_{T}=\frac{\left(R_{1}+R_{2}\right)*R_{3}}{R_{1}+R_{2}+R_{3}}$$

Meanwhile, to better illustrate the variation trend of the R_T_, the qualitative changes in the resistance of individual NR/GRs and NR/CNTs layers with strain are presented. The corresponding parallel resistance (R_F_) and the resistance of our micro‐wrinkled Janus films (R_T_) were compared in Figure 3e–g.

In the initial state, microcracks can be observed in the wrinkles of the NR/GRs layer (Figure 3a, a_1_). Under low strain (0–100%), the gradual relaxation of wrinkles triggers the closure of microcracks, and the GR fillers change from compressed contact at the wrinkles to uniform contact after flattening, leading to an increase in the number of conductive paths and decreased R_2_ (Figure 3b, b_1,_ b_2_). Simultaneously, the conductive pathways under stretching are reduced in unwrinkled regions of both the NR/GRs layer and NR/CNTs layer, which leads to the increase in R_1_ and R_3_ (Figure 3b_3_). Owing to a lower resistance in the NR/GRs layer with a higher filler content compared to the NR/CNTs layer, the R_T_ mainly depends on the NR/GRs layer resistance in the low strain range (Figure 3b_3_, e). As a result, the decrease in R_2_ effectively suppresses the sharp rise in R_T_. Evidently, the obtained R_T_ after being connected in parallel is closer to a linear increase, which conforms to the fitting parallel resistance R_F_ (Figure 3e). As the strain increases from 100% to 200%, the wrinkles are completely flattened, and directional cracks begin to appear in the upper layer, linearly increasing R_1_ and R_2_ (Figure 3c, c_1,_ c_2_). In the lower layer, the spacing of CNTs increases as the elastomer stretches, leading to the disruption of continued network and a slow increase in R_3_ (Figure 3c_3_). At this time, the resistance of the NR/GR layer still primarily determines R_T_ (Figure 3f). Hence, the simultaneous slow increase of all resistance components synergistically enhances R_T_, resulting in high sensitivity and linearity (Figure 3f). At 200–300% strain, distinct discrepancies emerge between R_T_ and R_F_ can be observed (Figure 3g). The NR/GRs layer inherently has low strain tolerance. But R_T_ benefits from strong interfacial interactions between NR/GRs and NR/CNTs layers, which boost interlayer stress transfer and enable coordinated deformation of the two layers. Moreover, this robust interfacial effect makes the bilayer better adapt to large deformations. In contrast, R_F_ only reflects a single‐layer resistance response, failing to capture the synergistic deformation enabled by the bilayer's strong interfaces. Hence, R_F_ shows a distinct resistance trend (e.g., a steeper increase) compared to R_T_. At this high strain, agglomerated structures in the CNTs network are stretched under large strain, causing the conductive paths to undergo discontinuous fracture and reconstruction, thus resulting in a continuous linear increase in R_3_ (Figure 3g) [36, 37]. In the NR/GRs layer, the *π–π* interactions between GR sheets are disrupted, and the conductive network undergoes large‐area delamination, resulting in an abrupt resistance jump [38, 39]. Eventually, the NR/GRs layer within the Janus film breaks under such large strain due to the stress concentration behind the wrinkled region in the NR/GRs layer, leading to its failure (Figure 3d_3_), which can be clearly observed in a resistance cutoff for the practical NR/GRs layer (Figure 3g). Owing to the good interfacial interaction between NR/CNTs and NR/GRs layers, electrons can still be transported in the Janus structure. The lower‐layer CNTs network serves as the dominant component. The resistance (R_3_) increases linearly with strain, thus ensuring the maintenance of a linear response even under high strain, as depicted in the R_T_ curve (Figure 3g). Therefore, the Janus structure not only enhances linearity but also expands the strain‐sensing range.

Overall, the sensing mechanism can be attributed to the following three points. First, the wrinkled structure of the upper NR/GRs forms a stress‐guided channel during the straining process. When subjected to external force, cracks do not propagate randomly; instead, they extend orderly and progressively along the direction of the wrinkles. As strain increases, the length and number of cracks increase linearly, and correspondingly, the upper‐layer resistance (R_2_) rises uniformly. Besides, strain‐phase segmented response enables smooth transition across the full strain range, which endows the sensor's resistance variation with a three‐stage linear segmentation. Furthermore, the synergistic effect of upper and lower parallel resistances eliminates nonlinear abrupt changes of resistance.

### 2.3 Sensing Performance

**FIGURE 4:**
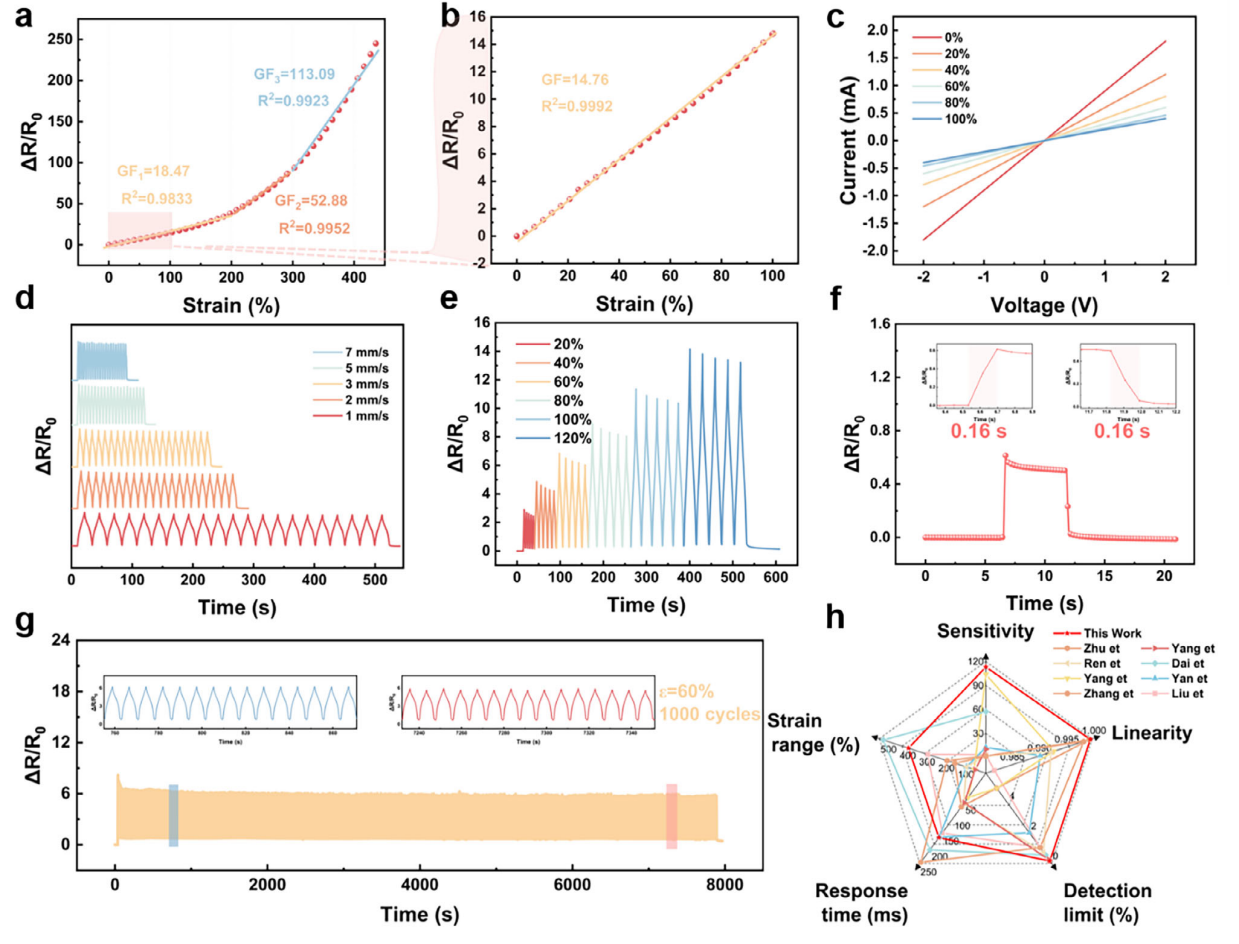
Strain sensing performance of the micro‐wrinkled Janus structured NR/CNTs@NR/GRs film with 60 phr GRs. (a) Relative resistance response and GF of the sensor over a wide strain range. (b) Relative resistance variations of the sensor under 100 % tensile strain. (c) Volt‐current characteristic curve of the sensor under different tensile strains. (d) The relative resistance response of the sensor under 60% strain at different strain speeds. (e) The relative resistance response at a speed of 100 mm/min at 20–120% strain. (f) Response/recovery time of the sensor at 1% strain. (g) Real‐time resistance changes during 10,00 cyclic strain tests under 60% strain. (h) Comprehensive performance comparison of our prepared sensors with other reported works.

Based on the ordered regulation of wrinkled structures, precise connection of segmented responses, and complementary effect of the parallel circuit, the designed sensor shows significantly improved sensing linearity and sensitivity. As depicted in Figure S12, the sensor with 60 phr GRs exhibits the optimized resistance response. Specifically, the sensor exhibits a monotonic increase in ΔR/R_0_ up to its failure strain of ∼450% (Figure 4a). The resistance‐strain relationship can be divided into three linear regions with gauge factors (GF) of 18.47 (0–200%), 52.88 (200–300%), and 113.09 (300–450%), highlighting its exceptional sensitivity across a wide strain range [40, 41, 42]. Remarkably, the sensor maintains an ultra‐high linearity (R^2^ > 0.999) within 100% strain (Figure 4b; Figure S13). Current‐voltage measurements from –2 to 2 V confirm standard ohmic behavior (Figure 4c), indicating a stable and well‐formed conductive network. The measurable hysteresis behavior in the resistance‐strain response of this sensor can be observed in Figure S14. Quantitatively, the largest hysteresis magnitude of this sensor is approximately 20% at a strain, indicating a slight deviation between the sensor's resistance response during strain loading vs. unloading.

The sensor also demonstrates outstanding stability, with negligible resistance variation under different tensile rates at a fixed 60% strain (Figure 4d). Dynamic cycling tests at strains ranging from 1% to 300% show regular and repeatable electrical signals (Figure 4e; Figure S15). Moreover, the sensor detects subtle deformations, exhibiting a resistance response at 0.1% strain (Figure S16), and achieves a rapid response time of 0.16 s under 1% strain at 1000 mm/min (Figure 4f). Long‐term reliability is confirmed through 1000 stretching‐releasing cycles at 60% strain, with no significant degradation in performance (Figure 4g). Meanwhile, post‐cycling SEM characterizations of the sensor confirm that the wrinkling morphology remains largely unchanged without structural damage or irreversible deformation (Figure S17). The wrinkled structure formed by pre‐stretching possesses excellent structural stability against cyclic deformation, providing powerful evidence that the sensor maintains stable performance without significant shifts. Taken altogether, our micro‐wrinkled Janus‐structured strain sensor integrates high sensitivity, broad strain range, excellent linearity, low detection limit, and fast response, outperforming most previously reported conductive composites (Figure 4h; Table S1) [15, 21, 43, 44, 45, 46, 47, 48].

### 2.4 Application in Human Motion Detection and Human‐Computer Interaction

**FIGURE 5:**
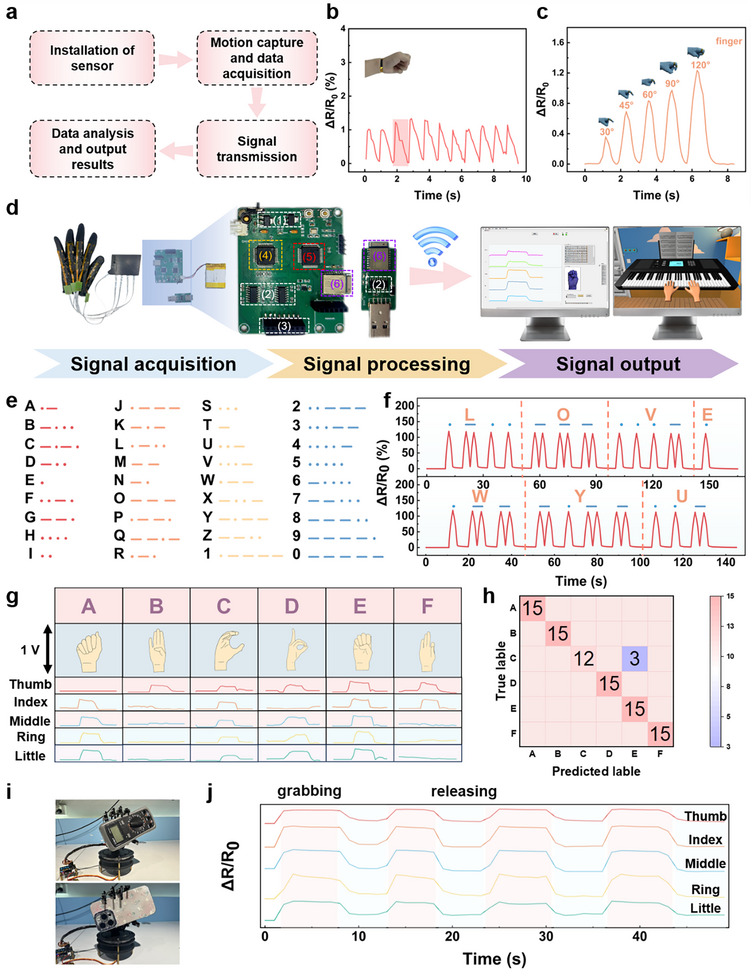
The application of the sensor in human motion detection, intelligent perception, and recognition. (a) Schematic diagram of the signal transmission process. (b), (c) The signal output for pulse and finger bending. (d) Schematic diagram of the intelligent glove, including the main hardware circuit structure for gesture recognition and human‐computer interaction. (e) Morse code sequences corresponding to 26 English letters and 10 Arabic numerals. (f) Coding examples of “LOVE” and “WYU” are represented by Morse code. (g) Typical gestures and the corresponding signal outputs. (h) Confusion matrix for gesture recognition results of A to F. (i) Photographic illustrations of the mechanical hand grasping a multimeter and a smartphone, respectively. (j) The electric signals for the machine hand's grasping and releasing actions.

In light of the excellent mechanical properties, electric conductivity, wide linear range, sensitivity, and facile process, the micro‐wrinkled Janus structured film exhibits a promising application perspective as a flexible strain sensor used in human motion detection and intelligent perception and recognition. First, we placed the sensor on the key part of the body for real‐time physiological signal detection. Due to its low detection limit and wide detection range, the sensor can detect and convert strain into electric signals for full‐range human movements—ranging from small movements (e.g., pulses) to large ones (e.g., joint movements). These signals are collected by the circuit acquisition board, then transmitted to a host computer via a Bluetooth module for subsequent data analysis and processing (Figure 5a). For example, when the sensor is attached to a volunteer's wrist, the sensor can capture subtle pulse signals, providing valuable information for real‐time pulse monitoring devices in athletic scenarios and home health management devices for hypertensive patients (Figure 5b). Moreover, the sensor can detect swallowing motions when mounted on the throat (Figure S18) and recognizes subtle facial expressions such as cheek puffing (Figure S19) and smiling (Figure S20) in real time. Furthermore, the sensor exhibits reliable performance in monitoring joint movements. When fixed on a finger, it generates reproducible resistance signals that correlate with different bending angles, demonstrating its ability to quantify the degree of articulation (Figure 5c). Similar responsiveness is observed when the sensor is deployed on the wrist, elbow, knee, and abdomen, allowing for the evaluation of movement speed and breathing patterns (Figure S21). Furthermore, to demonstrate our sensor's large‐strain applicability, we integrated it into a knee brace to monitor walking and running. During walking, it reliably captures stable resistance signals matching the large strain from knee flexion/extension (Figure S22a). For running, it also records clear, responsive signals corresponding to the knee joint's large‐strain changes (Figure S22b).

It's well known that high linearity is a key prerequisite for sensors to be efficiently integrated into circuits and stably drive devices. The obtained micro‐wrinkled Janus‐structured strain sensor exhibits excellent linear response, which can eliminate the need for compensation units in the signal processing circuit and thus simplify the hardware design of sensor‐circuit integration. Besides, this high linearity facilitates one‐to‐one mapping between strain and electrical signals, greatly reducing the computational complexity for the host computer in processing interactive motion data and thereby ensuring full‐range motions are accurately converted into control commands. When applied to human‐computer interaction scenarios, the sensor fully leverages its linearity advantages, effectively enhancing the response speed and recognition accuracy of interactive commands. Therefore, thanks to its high linear response range, an intelligent wireless glove system based on the designed sensor was successfully developed to validate its application potential in future intelligent perception and recognition. Here, the well‐defined system consists of the micro‐wrinkled Janus structured strain sensors, a circuit system (1–3), a signal acquisition chip (4), a main control unit (MCU, 5), a Bluetooth data transceiver module (6), and a host computer (Figure 5d). Among them, the sensors were fixed at the finger joints of the glove to collect strain signals generated during finger bending. Besides, analog switches were employed to manage the connection status of the sensors, allowing multiple sensors to be linked to the MCU for dynamic channel switching and multi‐channel data acquisition. The resistance output by the sensor is first converted into a voltage signal via an operational amplifier in the circuit system, and then sampled by the AD7606 analog‐to‐digital conversion chip and transmitted to the MCU for preliminary processing. Finally, the processed data is wirelessly transmitted via the Bluetooth module to the host computer for further analysis and visualization, such as gesture recognition and human‐computer interaction (Figure 5d). Figure 5e shows Morse code sequences corresponding to 26 English letters and 10 Arabic numerals, where rapid finger bending and double bending represent “dots” and “dashes” in Morse code, respectively. The electrical signals generated by continuous finger bending actions successfully expressed letter combinations such as “LOVE” and “WYU” (Figure 5f). In addition, the system demonstrates the capability to recognize more complex human movement patterns. For example, gesture recognition experiments were conducted using the first six English letters (A–F) as typical representatives. Figure 5g presents photographic depictions of each letter‐specific gesture and their corresponding five‐finger strain signal profiles. The results indicate that different gestures exhibit distinctively discriminable signal waveforms, highlighting the system's effectiveness in distinguishing between unique movement patterns. Moreover, the confusion matrix depicted in Figure 5h reveals that the glove achieves remarkably high accuracy in gesture classification tasks, validating the sensor's application potential in gesture recognition (Video S1). To further validate the application potential of the sensor in the field of human‐computer interaction, a piano mini‐game was developed, where volunteers can complete piano‐playing operations by wearing the intelligent glove (Video S2). This result fully demonstrates the feasibility and practicality of the sensor in interactive control scenarios. Furthermore, the intelligent glove was applied to a mechanical hand control system (Video S3). Figure 5i illustrates the process of the mechanical hand grasping a multimeter and a smartphone, while Figure 5j shows the resistance signal changes of the five fingers during the mechanical hand's grasping and releasing actions. The stable resistance output is observed during the grasping‐releasing process. The above results further verify that the intelligent glove based on this sensor demonstrates excellent performance in various human‐computer interaction applications, possessing good practical value and promotion potential.

## 3 Conclusions

In summary, a bio‐inspired Janus NR/GRs@NR/CNTs film with a wrinkle‐crack structure was fabricated through a simple layer‐by‐layer vacuum filtration method followed by a pre‐stretching strategy. Benefiting from the heterogeneous conductive network and wrinkle feature, the modulus and electron transport manner of the NR/GRs and NR/CNTs layers were tuned. The wrinkled structure of high‐modulus NR/GRs forms a stress‐guided channel and facilitates the linear increase in both the length and density of cracks, thus preventing the rapid decrease in electric conductivity of the Janus film. Besides, strain‐phase segmented response enables smooth transition across the full strain range, which endows the sensor's resistance variation with a three‐stage linear segmentation. Furthermore, the synergistic effect of upper and lower parallel resistances eliminates nonlinear abrupt changes of resistance. As a result, this Janus elastomeric sensor exhibits ultra‐high linearity (R^2^ > 0.999) and excellent sensitivity (GF > 14) within the full‐scale strain range of 100% strain. Meanwhile, this sensor has a wide detection range (>400%), an extremely low detection limit of 0.1% strain, fast response/recovery times of 0.16/0.16 s, and outstanding repeatability and stability. ​ The micro‐wrinkled Janus structured strain sensor has been successfully demonstrated for human motion detection, intelligent perception, and recognition, showing great potential in areas such as smart wearable electronics and human‐machine interaction.

## 4 Experimental Section

### 4.1 Materials

Pre‐cured natural latex (approx. 60wt.%) was supplied by Thailand Three Trees International Holdings Co., Ltd. Graphene (Item No.:SH‐GR0991) was provided by Shenzhen Suiheng Technology Co., Ltd. Hydroxyethylated multi‐walled CNTs (MWCNTs, average length of 5–15 µm, diameter of 10–20 nm, purity of 97%) were acquired from Shenzhen Zhongsen Linghang Technology Co., Ltd. Triton X‐100 (AR, 99%) purchased from Adamas was used without further purification.

### 4.2 Preparation of NR/CNTs and NR/GRs Dispersions

First, the dispersion was prepared by dissolving 0.15 g of Triton X‐100 in 15 mL of water. Subsequently, 0.018 g of WCNTs and 0.2 g of NR latex were added to the dispersion and stirred thoroughly to obtain a uniform NR/CNTs dispersion. Similarly, 0.006, 0.018, 0.036, 0.048, and 0.06 g of GRs were added to the dispersion, including 0.15 g of Triton X‐100, respectively. Then, 0.1 g of NR latex was added to the different components of the conductive filler dispersion, respectively, and ultrasonication was performed for 30 min to ultimately obtain uniform NR/GRs dispersions. Besides, a pure NR dispersion was also prepared.

### 4.3 Preparation of Flexible Strain Sensors with Micro‐Wrinkled Janus Structure

First, the NR/CNTs film was prepared by vacuum filtration. Subsequently, NR/GRs dispersion was poured onto the primary film and filtered again to form a composite film with an asymmetric structure. The resulting composite films were dried in a vacuum oven at 70°C for 8 h to achieve full vulcanization. Then, the as‐prepared films were pre‐stretched at 200% strain to obtain micro‐wrinkled Janus‐structured films. The films were designated as NR/CNTs, NR/CNTs@NR/GRs‐10, NR/CNTs@NR/GRs‐30, NR/CNTs@NR/GRs‐60, NR/CNTs@NR/GRs‐80, and NR/CNTs@NR/GRs‐100, corresponding to GRs contents of 0, 10, 30, 60, 80, and 100 parts, respectively. One part indicates that there was 1 g of filler per 100 g of rubber in the NR/CNTs or NR/GRs layer. Finally, the films were prepared as sensors by connecting the two copper electrodes using silver paste.

### 4.4 Characterization

Scanning electron microscopy (SEM, FEI, NoVoTM nano SEM 430) was carried out to study the composite morphologies of as‐prepared micro‐wrinkle Janus structured films. The structural characterization of the obtained samples was analyzed by Fourier transform infrared (FT‐IR, Bruker Vector 70) and X‐ray diffraction (XRD, PANalytical B.V., X'Pert^3^Powder).

The dimensions and fixture holding distances of the strain transducers of the micro‐wrinkle Janus structure CECs were 20 mm × 5 mm and 20 mm, respectively. Two electrodes made of copper sheets at each end of the transducers were connected to a copper wire via silver paste for mechanical and sensing tests. The mechanical properties were tested using a tensile tester (TestometricX250‐1, Britain) with a tensile rate of 100 mm/min. real‐time resistance of the NR/m‐MWCNTs CECs strain transducers under various tensile deformations was recorded by means of a digital multimeter (Keithley 2450, US).

The mechanical simulation analysis of the micro‐wrinkled Janus film was evaluated by ANSYS software. A representative volume element (RVE) model of the target microstructural architecture was constructed using SolidWorks to characterize the features of its periodic arrangement, and the model was subsequently imported into the Ansys Workbench platform for static tensile simulation. In the Engineering Data module, material constitutive models incorporating Young's modulus, yield strength, and uniaxial tensile stress–strain curves were established separately for each component in different regions of the RVE and mapped to the corresponding geometric regions. In the Mesh module, solid elements suitable for micromechanical analysis were selected, and high‐quality mesh discretization was achieved by controlling mesh size and element quality metrics (e.g., aspect ratio, skewness). In the Static Structural module, periodic boundary conditions were applied to the RVE to maintain its microstructural periodic deformation characteristics, with a uniaxial tensile displacement load imposed on one end and the corresponding directional degree of freedom constrained on the other end. After solving, the stress distribution in microregions under various strain levels was extracted and visualized in the Solution module to reveal the micromechanical behavior of the structure during tensile deformation.

To achieve accurate gesture recognition, the following data processing and modeling workflow was adopted for the collected sensor data. First, the peak signal features generated by gesture‐specific finger‐bending movements were extracted to complete feature engineering for the raw data. Next, the dataset was split into an 85/15 ratio, with the two subsets serving as the model's training set and test set, respectively. The K‐Nearest Neighbors (KNN) algorithm was selected as the classification model, where the training set data was fed into the model for parameter learning and training, followed by evaluating the model's classification performance using the test set data. According to the results of the confusion matrix, the model's recognition accuracy for different gestures can be obtained.

### 4.5 Ethical Statement

All experiments were performed in accordance with all local laws and approved by all relevant ethics bodies. Informed signed consent was received from the participants of the experiments with the wearable sensor devices.

## Conflicts of Interest

The authors declare no conflicts of interest.

## Supporting information




**Supporting File 1**: advs74018‐sup‐0001‐Movie S1.mp4.

**Supporting File 2**: advs74018‐sup‐0002‐Movie S2.mp4.

**Supporting File 3**: advs74018‐sup‐0003‐Movie S3.mp4.

**Supporting File 4**: advs74018‐sup‐0004‐SuppMat.docx.

## Data Availability

The data that support the findings of this study are available in the supplementary material of this article.
