# Supplementary material for: Ultrahigh‐Linear Bio‐Inspired Janus Elastomeric Strain Sensor with High Sensitivity and Stretchability via Surface Wrinkle Engineering
Source: Adv Sci (Weinh). 2026 Jan 27;13(19):e24269. doi: 10.1002/advs.202524269 (PMC13045355; doi:10.1002/advs.202524269)
Supplement: Supplementary file 4 — Supporting File 4: advs74018‐sup‐0004‐SuppMat.docx. [file ADVS-13-e24269-s001.docx]

**Ultrahigh-Linear Bio-Inspired Janus Elastomeric Strain Sensor with High Sensitivity and Stretchability via Surface Wrinkle Engineering**

Jing Lin^a, *^, Ye Li^a^, Simi Yu^a^, Xing Cheng^a^, Longxin Qian^a^, Yufei Chen^a^, Womin Li^a^, Zhipeng Yang^b^, Yinlei Lin^b^, Dechao Hu^b, *^, Jianyi Luo ^a, *^, Lan Liu^c, *^

^a^ *Research Center of Flexible Sensing Materials and Devices, School of Applied Physics and Materials, Wuyi University, Jiangmen 529020, China*

^b^ *School of Materials and Energy, Foshan University, Foshan 528000, China*

*^c^* *Key Lab of Guangdong High Property and Functional Macromolecular Materials, School of Materials Science and Engineering, South China University of Technology, Guangzhou 510640, China*

* **Corresponding author**

^*^ E-mail address: [Jinglin@wyu.edu.cn](mailto:Jinglin@wyu.edu.cn) (J. Lin), [msdchu@fosu.edu.cn](mailto:msdchu@fosu.edu.cn) (D. Hu), [luojiany@wyu.edu.cn](mailto:luojiany@wyu.edu.cn) (J. Luo), [psliulan@scut.edu.cn](mailto:psliulan@scut.edu.cn) (L. Liu)


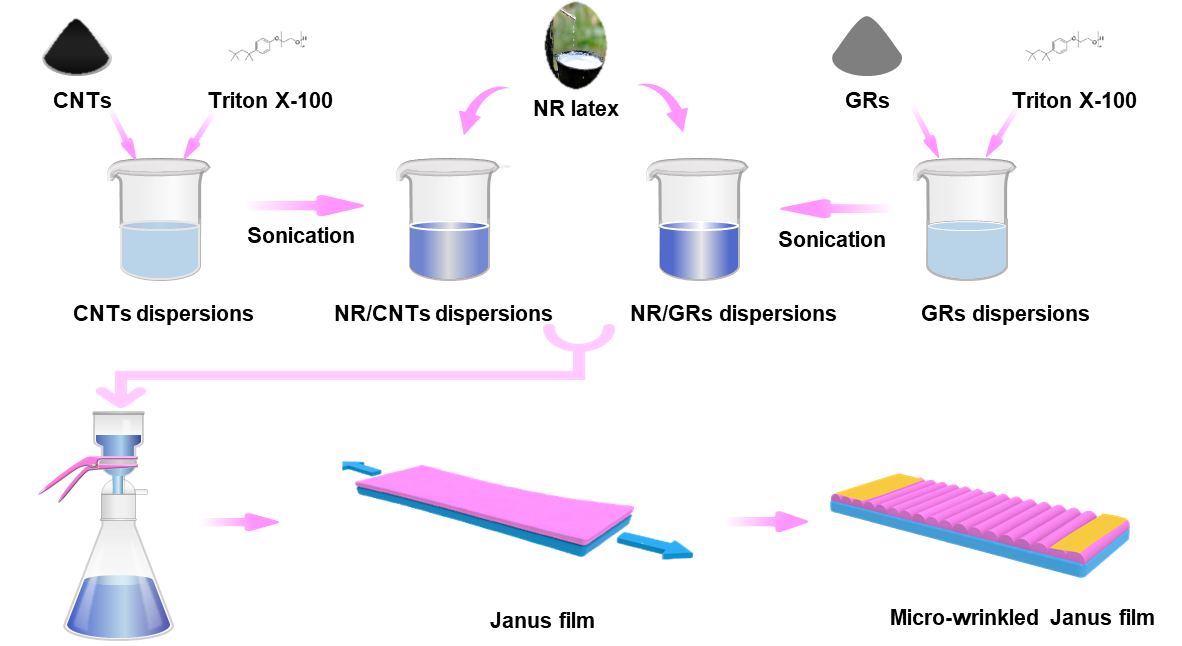
 **Figure S1.** Fabrication schematic illustration of the micro-wrinkled Janus film.


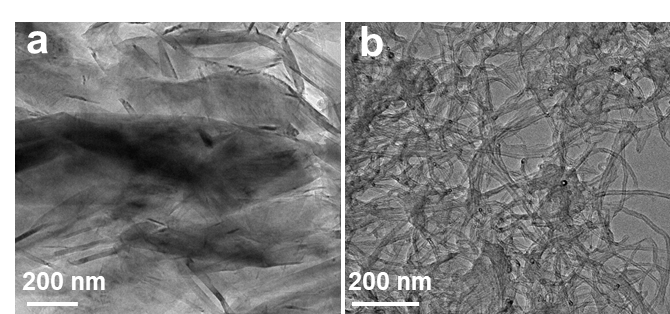


**Figure S2.** TEM images of a) NR/GRs layer and b) NR/CNTs layer for the micro-wrinkled Janus film.


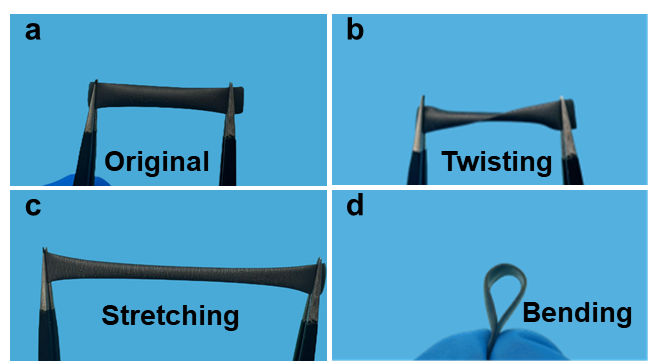


**Figure S3.** a-d) Photographs of the micro-wrinkled Janus film under twisting, stretching, and bending.


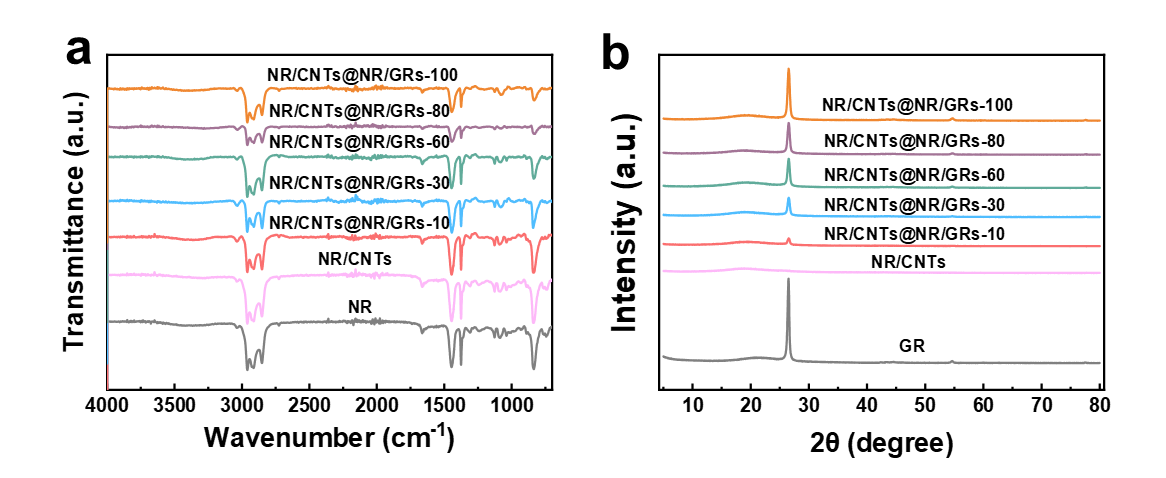


**Figure S4.** a) FTIR and b) XRD spectra of NR/CNTs and the micro-wrinkled Janus film with different content of GRs.

**
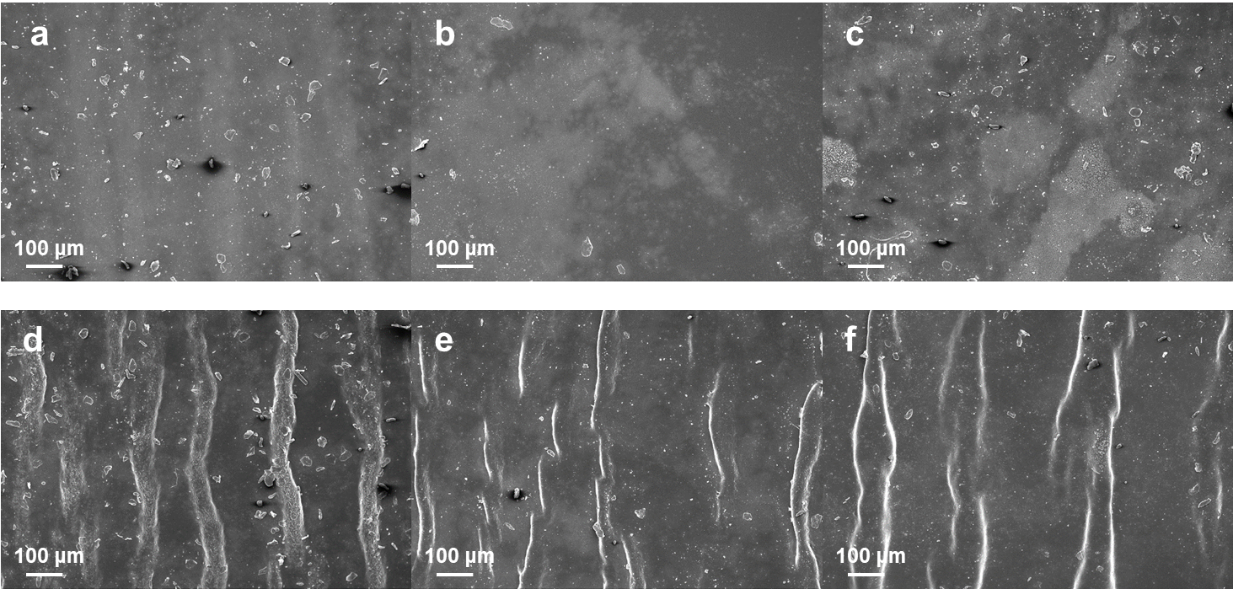
**

**Figure S5.** Surface SEM images of the micro-wrinkled Janus films with of a) 0, b) 10, c) 30, d) 60, e) 80, f) 100 phr graphene after pre-stretching of 200% strain.





**Figure S6.** Typical stress-strain curves for the micro-wrinkled Janus film with GRs of 60 phr after different pre-stretching conditions.





**Figure S7.** The resistance response curves of the micro-wrinkled Janus film with GRs of 60 phr after different pre-stretching conditions.

**

**

**Figure S8.** Linear fitting curves of the micro-wrinkled Janus film with GRs of 60 phr after different pre-stretching conditions under 100% strain.


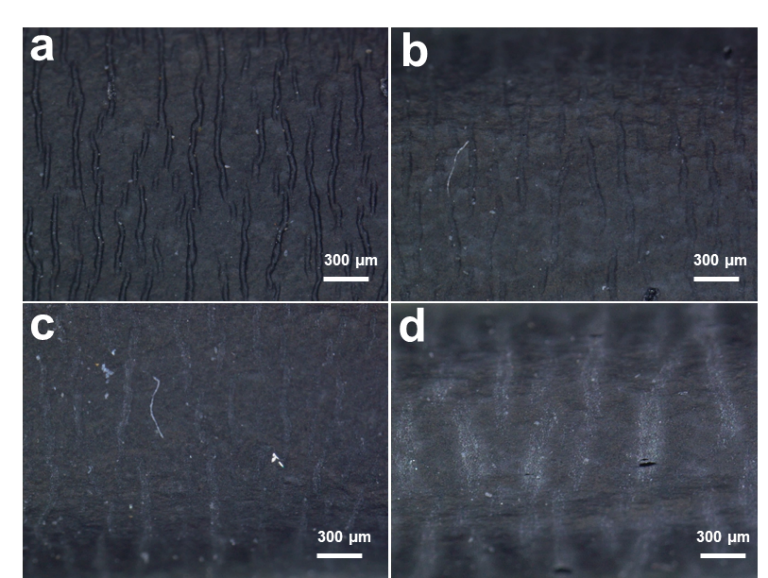


**Figure S9.** Leica camera images of cracks for the micro-wrinkled Janus films at different strain: a) 0%, b) 100%, c) 200%, and d) 300% strains.


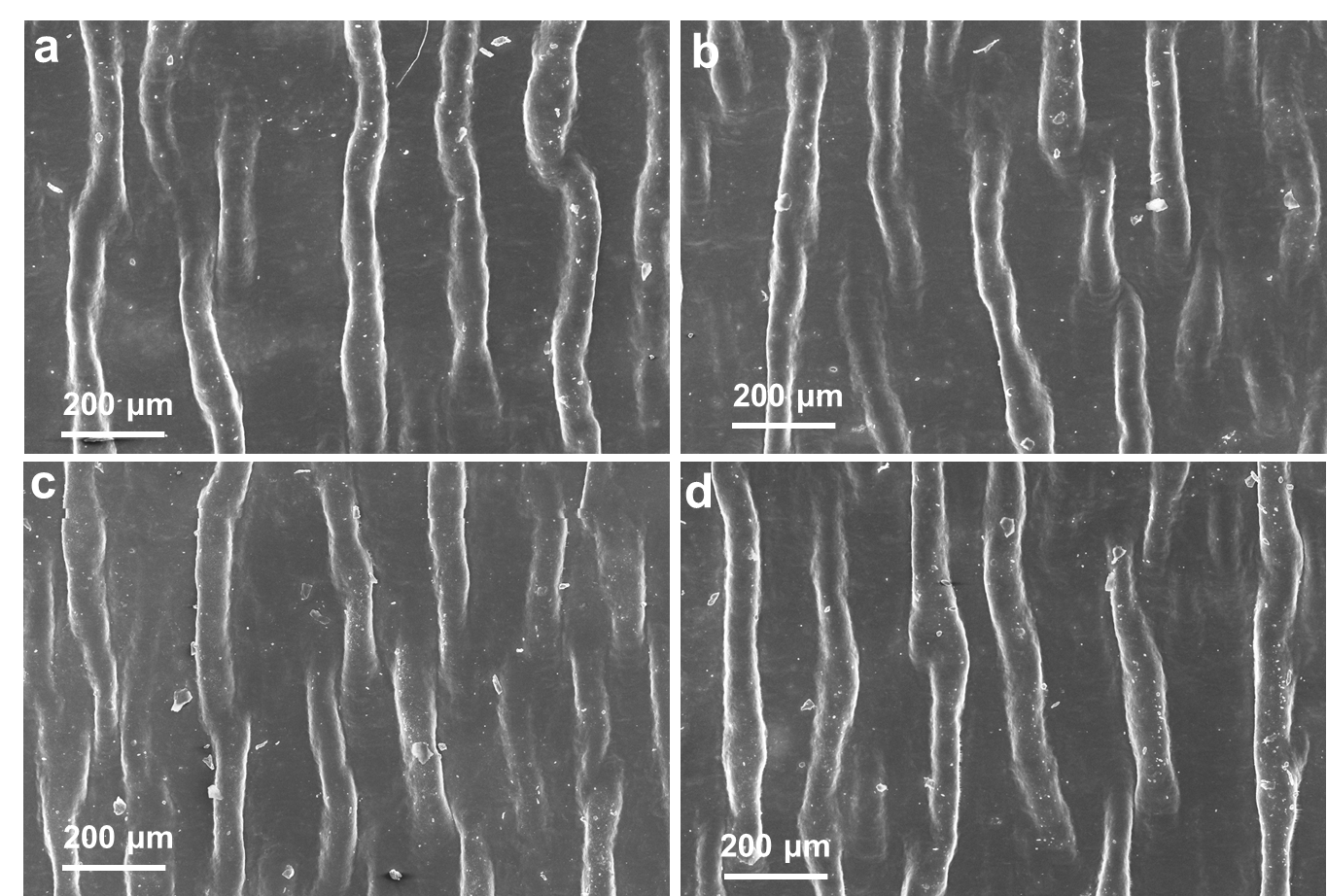


**Figure S10.** Surface SEM images of the micro-wrinkled Janus films under different temperature and humidity: a) 25 ^o^C, 30% RH, b) 25 ^o^C, 60% RH, c) 50 ^o^C, 30% RH, and d) 50 ^o^C, 60% RH.





**Figure S11.** Conductivity of the micro-wrinkled Janus film with different GRs contents.

**Figure S12.** The relative resistance response of the micro-wrinkled Janus film-based sensors with different GRs contents.


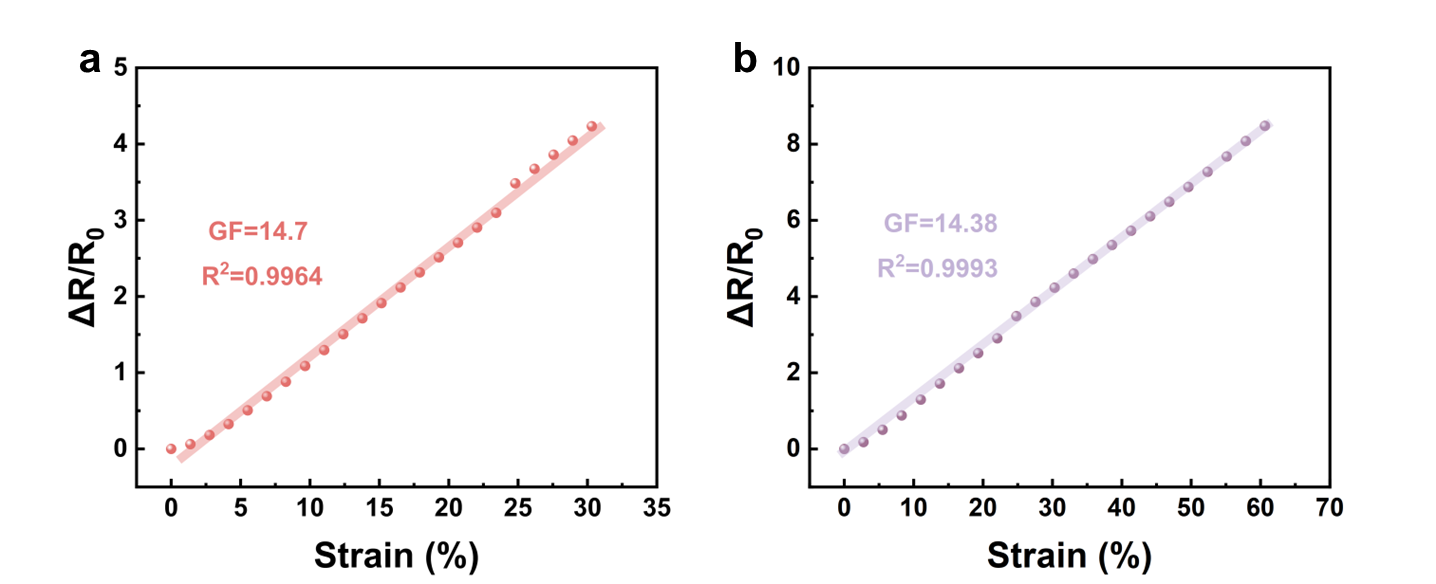


**Figure S13.** Linear fitting curves under a) 0-35% and b) 0-70% strain of the micro-wrinkled Janus film-based sensors with 60 phr GRs under different strains.


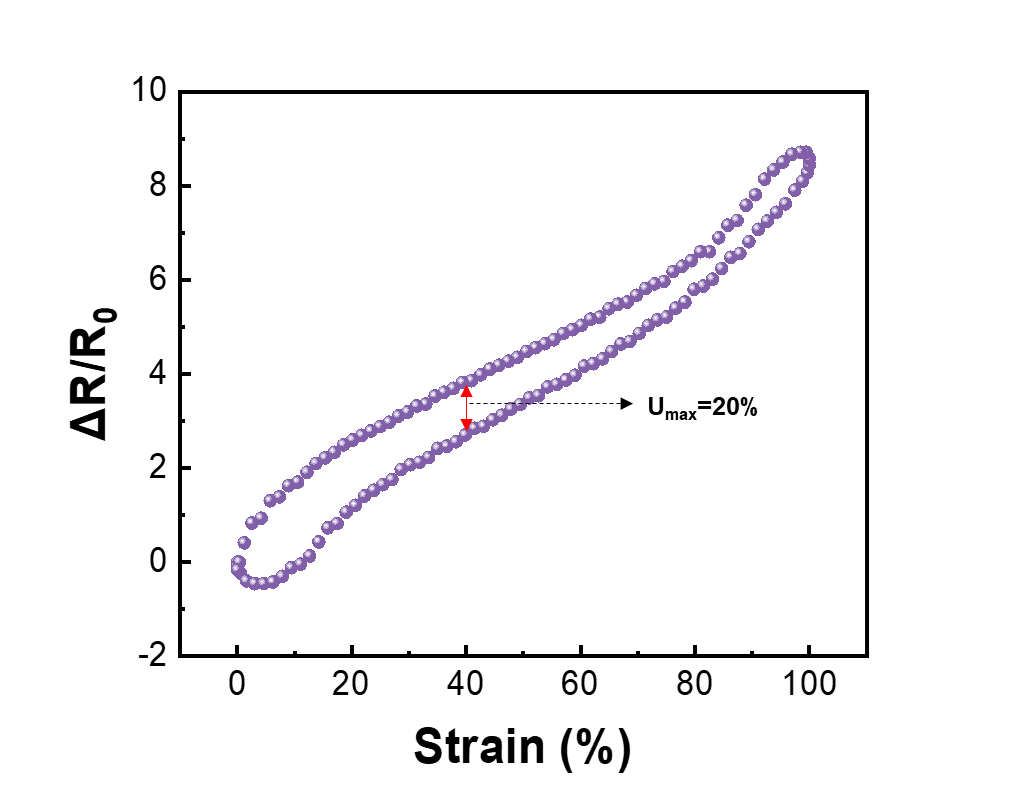


**Figure S14.** Hysteresis curves of the micro-wrinkled Janus film-based sensors at 100% strain.


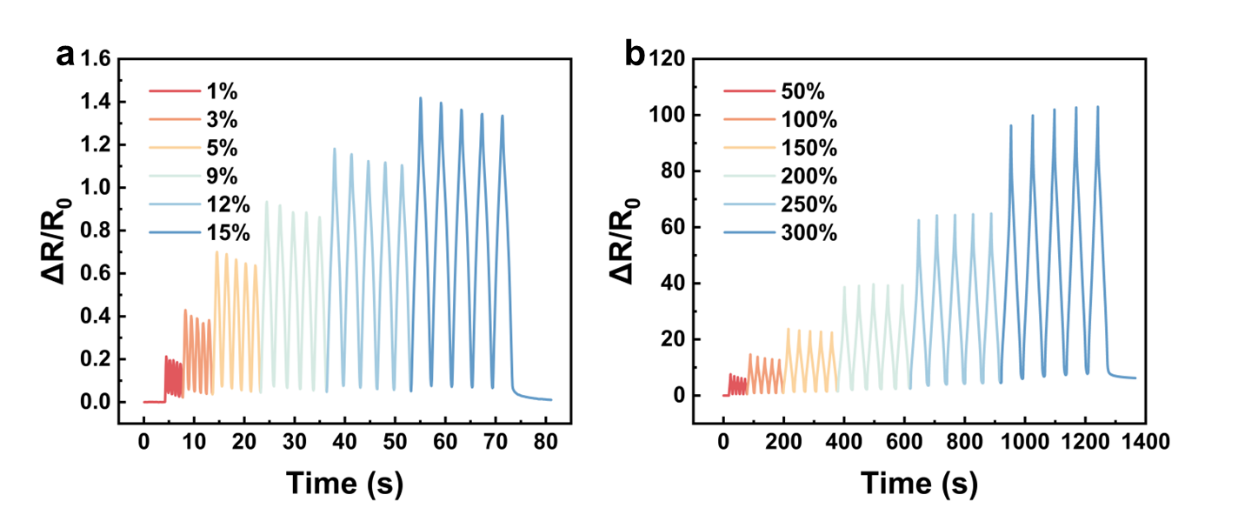


**Figure S15.** Resistive response of the micro-wrinkled Janus film-based sensors with 60 phr GRs under a) small and b) large strain.





**Figure S16.** Resistive response of the micro-wrinkled Janus film-based sensors with 60 phr GRs under small strain.

**
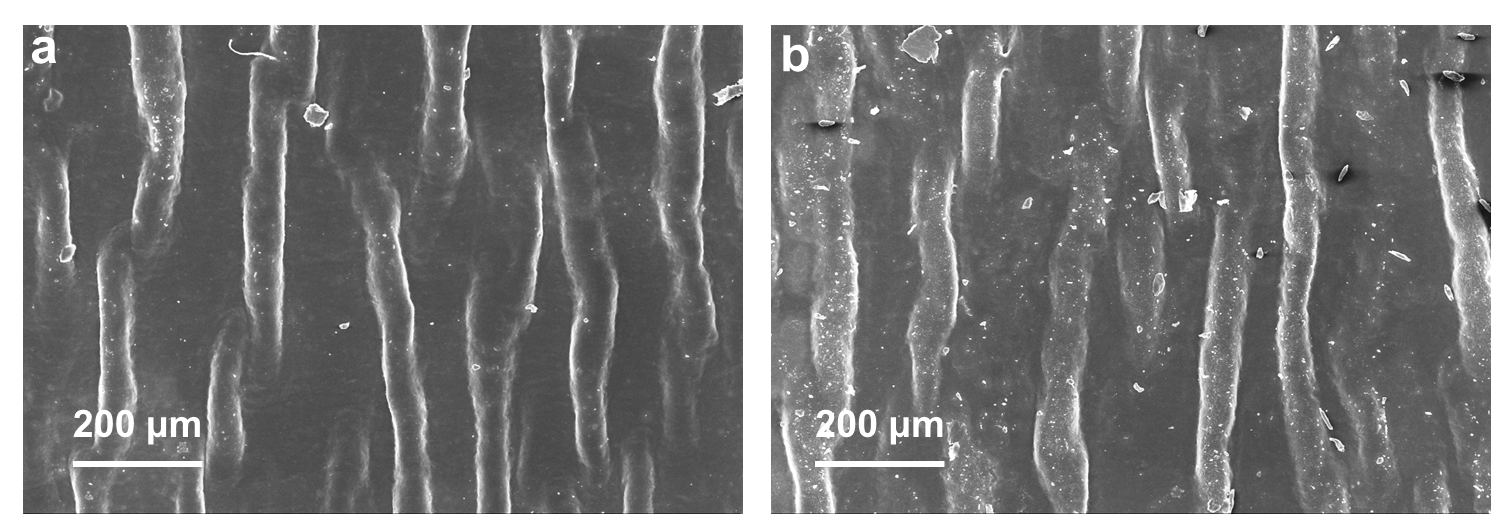
**

**Figure S17.** SEM images of the micro-wrinkled Janus film-based sensors with 60 phr GRs a) before and b) after cycles test at 60% strain.

**
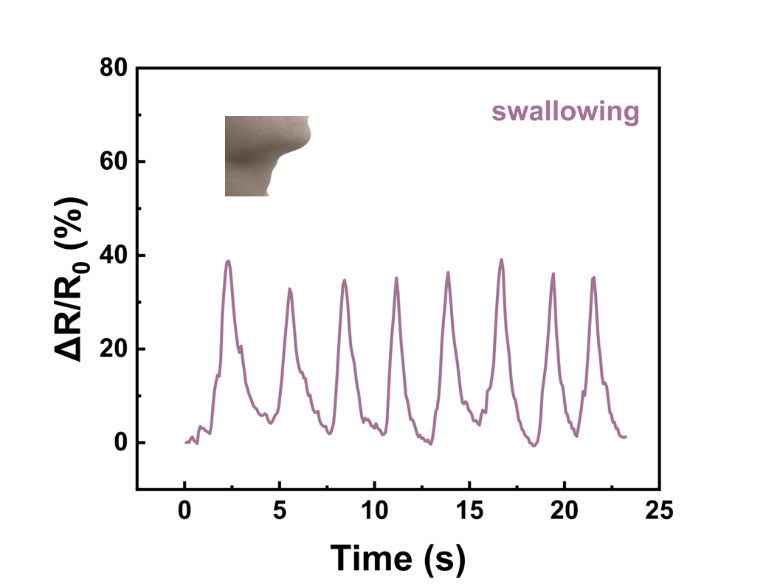
**

**Figure S18.** The signal output of the sensor for the human body's swallowing action.


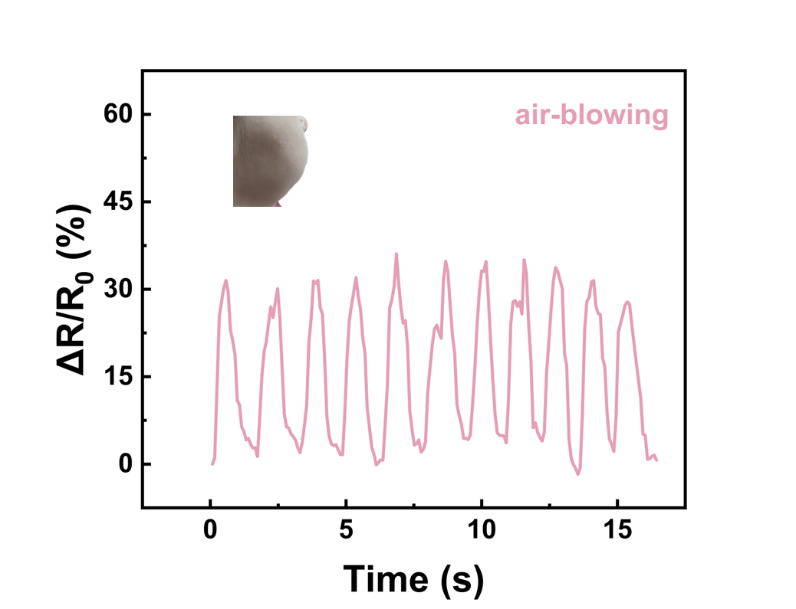


**Figure S19.** The signal output of the sensor for the blowing action.

**
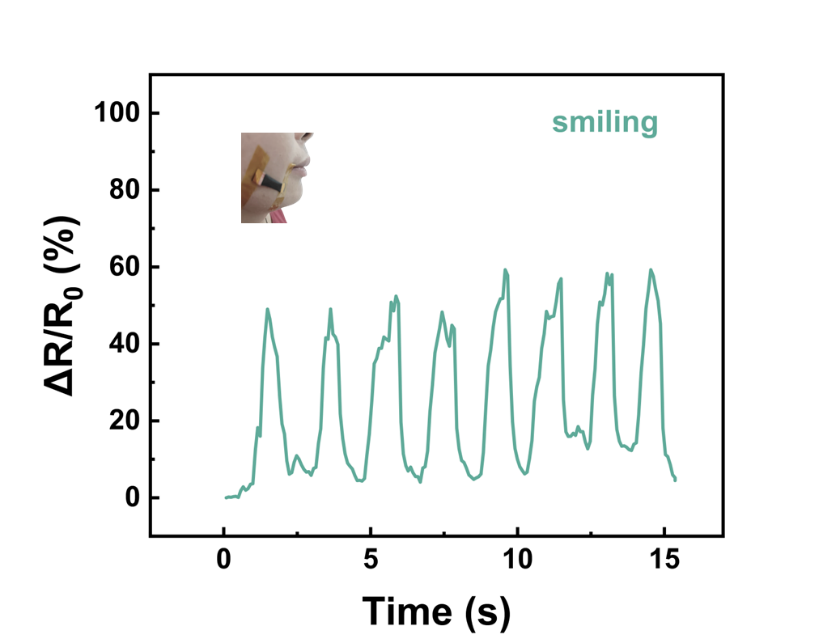
**

**Figure S20.** The signal output of the sensor for facial expressions.

**
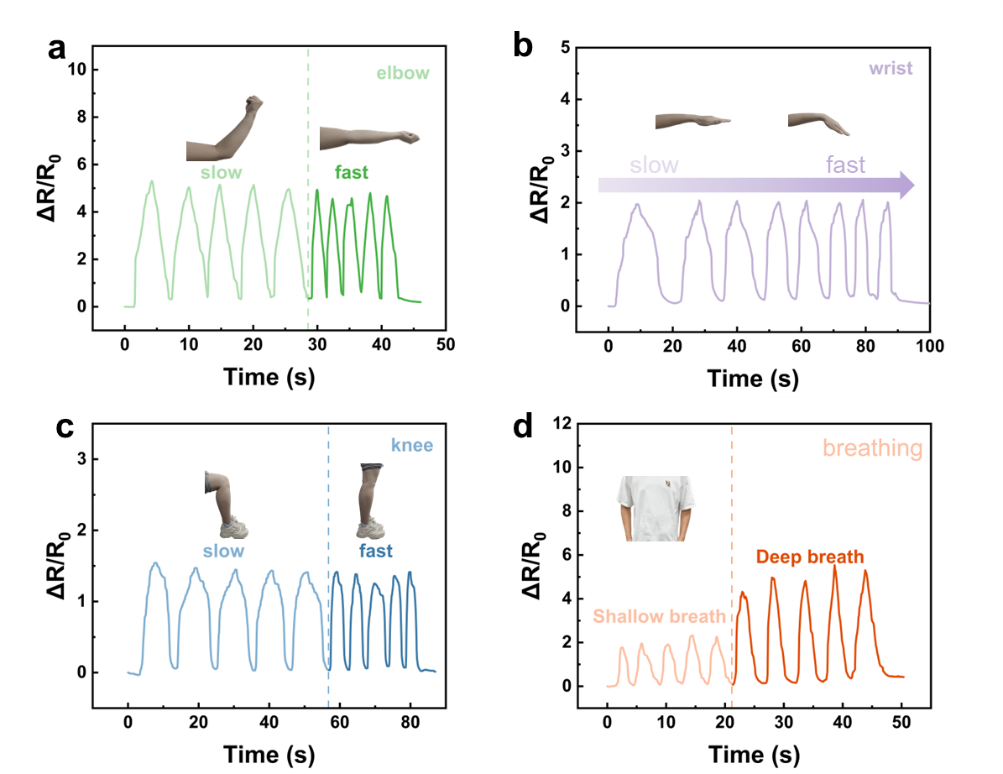
**

**Figure S21.** The signal output of the sensor attached on a) the elbow, b) wrist, c) knee, and d) abdomen of the human body for different frequencies of movement.

**
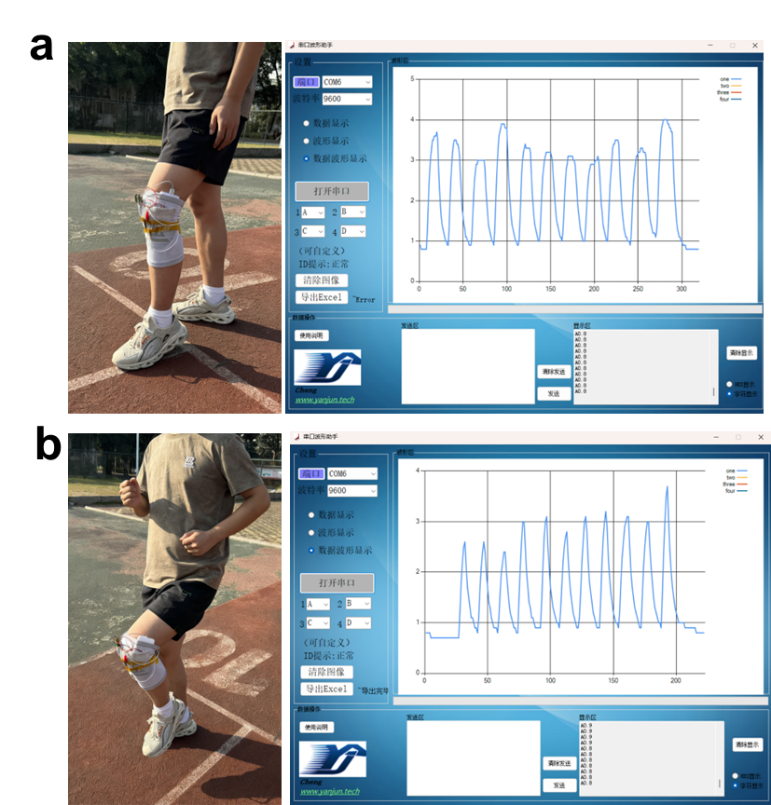
**

**Figure S22.** The signal output of the sensor integrated it into a knee brace to monitor a) walking and b) running.

**Table S1.** Comprehensive comparison of the strain sensing performances of our prepared sensor with other reported works.

| Sensor system | Sensitivity | linearity | Detection limit (%) | Response time (ms) | Strain range (%) | Ref. |
| --- | --- | --- | --- | --- | --- | --- |
| MXene/AgNWs/LM | 3.22 | 0.981 | 1 | 145 | 300 | ^[1]^ |
| PPM-NL | 1.12 | 0.999 | 5 | 60 | 160 | ^[2]^ |
| CNT/DTY | 12.43 | 0.990 | 2 | 158 | 100- | ^[3]^ |
| MXene/AgNW | 104 | 0.992 | 5 | 30 | 60 | ^[4]^ |
| TFMCF | 57.2 | - | 0.5 | 200 | 530 | ^[5]^ |
| CNTs/CBs/PDMS | 2 | 0.992 | 0.7 | 50 | 100 | ^[6]^ |
| SP/Ag | 10 | - | 0.2 | 46.9 | 55 | ^[7]^ |
| PVA/CA/AgNPs | 1.6 | 0.998 | 1 | 240 | 200 | ^[8]^ |
| NR/CNTs@NR/GRs | 113.09 | 0.999 | 0.1 | 160 | 400 | This work |

[1] Y. Wang, W. Qin, M. Yang, Z. Tian, W. Guo, J. Sun, X. Zhou, B. Fei, B. An, R. Sun, "High linearity, low hysteresis Ti3C2Tx MXene/AgNW/liquid metal self‐healing strain sensor modulated by dynamic disulfide and hydrogen bonds," *Advanced Functional Materials* 33 (2023): 2301587.

[2] S. Yuan, J. Bai, S. Li, N. Ma, S. Deng, H. Zhu, T. Li, T. Zhang, "A multifunctional and selective ionic flexible sensor with high environmental suitability for tactile perception," *Advanced Functional Materials* 34 (2024): 2309626.

[3] F. Huang, J. Hu, X. Yan, "A wide-linear-range and low-hysteresis resistive strain sensor made of double-threaded conductive yarn for human movement detection," *Journal of Materials Science & Technology* 172 (2024): 202.

[4] T. Wang, Z. Qiu, H. Li, H. Lu, Y. Gu, S. Zhu, G. S. Liu, B. R. Yang, "High sensitivity, wide linear‐range strain sensor based on MXene/AgNW composite film with hierarchical microcrack," *Small* 19 (2023): 2304033.

[5] X. Zhao, H. Guo, P. Ding, W. Zhai, C. Liu, C. Shen, K. Dai, "Hollow-porous fiber-shaped strain sensor with multiple wrinkle-crack microstructure for strain visualization and wind monitoring," *Nano Energy* 108 (2023): 108197.

[6] H.-B. Chen, Y. Ding, G. Zhu, Y. Liu, Q. Fang, X. Bai, Y. Zhao, X. Li, X. Huang, T. Zhang, B. Li, B. J. n. F. E. Sun, "A new route to fabricate flexible, breathable composites with advanced thermal management capability for wearable electronics," 7 (2023): 1.

[7] H. Q. Shao, K. D. Wei, T. Gong, J. Jia, C. Y. Tang, X. J. Zha, K. Ke, R. Y. Bao, K. Zhang, Y. Wang, "Elastic janus microarray film strain sensors with heterogeneous modulus and conductivity for healthcare and braille identification," *Advanced Functional Materials* 34 (2024): 2316134.

[8] L. Chen, X. Chang, H. Wang, J. Chen, Y. Zhu, "Stretchable and transparent multimodal electronic-skin sensors in detecting strain, temperature, and humidity," *Nano Energy* 96 (2022): 107077.
